# Handle with Care: A Narrative Review of Infant Safe Sleep Practices across Clinical Guidelines and Social Media to Reduce SIDS

**DOI:** 10.3390/children10081365

**Published:** 2023-08-09

**Authors:** Aysha Jawed, Catherine Ehrhardt, Molly Rye

**Affiliations:** 1 Johns Hopkins Children’s Center, Baltimore, MD 21287, USA; cehrhar2@jhmi.edu (C.E.); mrye1@jhmi.edu (M.R.); 2 Department of Pediatric Social Work, Johns Hopkins Children’s Center, Baltimore, MD 21287, USA; 3 Department of Pediatric Nursing, Johns Hopkins Children’s Center, Baltimore, MD 21287, USA

**Keywords:** social media, Facebook, Instagram, Twitter, sudden infant death syndrome, infant sleep-related deaths, American Academy of Pediatrics, Safe-to-Sleep

## Abstract

Sudden Infant Death Syndrome (SIDS) is a leading cause of infant mortality across the United States and the world. There are multiple environmental and behavioral determinants of sudden infant death which are modifiable risk factors and potential targets for intervention. In this increasingly digital era, health education and communication on SIDS have taken many forms, which extend to social media. Current published studies on coverage of infant safe sleep practices are scant and were published well before the newly revised guidelines of the American Academy of Pediatrics that review ways to prevent infant sleep-related deaths based on evidence-based SIDS-reduction measures. In this Perspective: Review of a Pediatric Field, the current state of published knowledge and coverage on a range of infant safe sleep considerations across social media are reviewed. We delineate gaps in the knowledge and practice as well as the central differences between the 2016 and 2022 AAP Safe Sleep guidelines. We also present recommendations for further research and practice which support coverage of future content on the revised guidelines across social media as the basis to present the most up-to-date and evidence-based information for reducing sudden infant death from sleep-related causes. Tapping into the potential of social media as a learning modality in health promotion also contributes towards the larger goal of the World Health Organization, United Nations International Children’s Emergency Fund (UNICEF), and Healthy People 2030 to reduce infant mortality on both global and national levels.

## 1. Introduction

Sudden Infant Death Syndrome (SIDS) is the sudden death of an infant less than one year of age which remains inconclusive following a comprehensive investigation involving a thorough autopsy, critical examination of circumstances surrounding infant death, and complete review of the clinical history [1,2]. SIDS remains the leading cause of death among infants between birth and 1 year of age [3]. Oftentimes, SIDS is referenced as crib death or cot death, given that causal factors are attributed to unsafe sleeping environments, which could involve a range of environmental and behavioral determinants [3]. Based on recently reported data from the American Academy of Pediatrics, there are approximately 3500 sudden unexpected deaths of infants (SUDI) each year in the United States (U.S.) that are inclusive of infant deaths from SIDS [4]. It is well evidenced that inflammation and infections also contribute to the incidence and prevalence of SIDS [5]. Specifically, viral infections of the respiratory tract are causal determinants of SIDS. In turn, respiratory infections represent more biological vulnerabilities in the Triple Risk Model that heighten the risk for SIDS. Many respiratory infections in SIDS cases involved inflammation of the airways and lungs of these infants [6,7,8].

In addition, pathophysiology of infant deaths from SIDS are also consistently attributed to abnormalities in both cardiac and respiratory control within the infant’s brainstem, thereby representing a constellation of biological vulnerabilities in the Triple Risk Model for SIDSs. These cardiac and respiratory complicating factors integrated with external states (e.g., sleep positioning, environmental tobacco exposure and thermal conditions) could heighten the infant’s risk for hypoxia, bradycardia, apnea and ultimately death [9,10]. In addition, nearly 10–15% of infant deaths from SIDS worldwide are likely attributed to cardiac arrythmias [11]. Furthermore, approximately one in 10 SIDS cases are correlated with mutations in the cardiac ion channel gene [12].

The Safe-to-Sleep campaign is at the heart of disseminating messages on safe sleep practices for infants across the United States. The campaign, which initially began in 1994 as the Back-to-Sleep campaign, is a multi-collaborative mass educational endeavor among the National Institute of Child Health and Human Development (NICHD), the American Academy of Pediatrics, the Maternal and Child Health Bureau of the Health Resources and Services Administration, First Candle, and the Association of SIDS and Infant Mortality Programs.

When the Back-to-Sleep campaign began in the earlier part of the 1990s, SIDS rates across the U.S. dropped by nearly 50%. In addition, the rate of prone sleeping position among infants also substantially declined by approximately 50% shortly following the inception of the Back-to-Sleep campaign [13]. However, for the past 27 years, since 1996, sudden unexpected death of infants (SUDI) rates have largely remained unchanged. Furthermore, during the first year of the COVID-19 pandemic, the SIDS rates increased by 15% across the U.S. with more than twice as many deaths among Black infants than White infants [14].

Oftentimes, campaigns have extended their reach and visibility beyond traditional sources of media to also disseminate content across nontraditional sources of media (e.g., social media). One of the benefits of social media is its global reach in delivering messages efficiently across the world. Based on a recent PEWS study, approximately 70% of adults in the U.S. utilize at least one social media platform [15]. Nearly 80% of these adults access YouTube, and 69% of them are connected to Facebook [15]. Instagram, Snapchat, and Twitter have a stronger following among adolescents and young adults [15]. In light of these figures during our digital era, it is crucial to account for social media as a method of communication and education for many populations given its prevalent usage and presence.

Since 2016, the American Academy of Pediatrics (AAP) taskforce has implemented significant changes on promoting safe sleep education, practices, and directions for further work to keep infants safe and sound in their sleep [16]. The newly revised AAP 2022 Safe Sleep guidelines present a range of evidence-based recommendations with more specifications on safe sleep surfaces, thermal conditions, tummy time and pacifier use, room-sharing and bed-sharing considerations, avoidance of additional substances, and usage of devices [4,17]. In addition, the guidelines build on existing research that has demonstrated racial and ethnic disparities in infant-sleep-related deaths as the basis to emphasize continued work in safe sleep education as part of achieving health equity across the infant population. Given the recency of these revisions, there have not been any published studies evaluating the existing coverage of content across social media platforms. However, published content on infant safe sleep across social media at this time precedes the 2022 AAP guidelines and in turn does not account for all of the up-to-date recommendations. Nevertheless, they are still points of intervention from prior coverage to build on for future work of coverage on social media of infant safe sleep practices and environmental determinants.

We review the key differences between the 2016 and 2022 AAP safe sleep guidelines as well as the existing literature on infant safe sleep across the most prominent social media platforms (Instagram, Facebook, and Twitter). There are no published studies to date on infant safe sleep content covered on YouTube, which forms a direction for our future endeavors. All three published studies involved conducting thematic and content analyses [18,19,20]. Formats of tweets in one study involved hashtags, messages and videos [18]. In another study examining content on Instagram, still images (photographs or video thumbnails) of either an infant sleeping or an infant sleep environment were assessed [19]. In a different study, posts and comments by mothers on Facebook were the sole formats examined for safe sleep content [20].

## 2. Primary Differences in Infant Safe Sleep Recommendations between the 2016 and 2022 AAP Guidelines

There are several key differences in the recommendations delineated in the AAP guidelines between 2016 and 2022. The 2022 AAP guidelines account for the growing tobacco epidemic, heath disparities and inequities in access to care and resources, education, and research. Although several of the recommendations have remained the same, there are multiple recommendations with expanded depth and breadth from the prior iteration that account for a range of evidence-based findings from research conducted over the years amidst development of the revised guidelines. Further, there is increased recognition of the misinterpretation of different monitoring devices that have persisted over time. In addition, the newly revised AAP 2022 guidelines recommend heightened media coverage of infant sleep-related content as an evidence-based SIDS-reduction measure [4].

Also in the AAP 2022 Safe Sleep guidelines, overheating prevention parameters clearly indicate the need to avoid usage of hats among infants except immediately following birth for the first couple of hours or during time in the NICU. In the 2016 AAP guidelines, overheating parameters were limited to room temperature considerations along with layering of clothing. Hats were not accounted for as a source of overheating for infants during time of sleep in the 2016 guidelines. Furthermore, there have been changes in the guidelines on parameters for dressing an infant during time of sleep. In the 2022 AAP guidelines, increased emphasis is placed on dressing an infant with light clothing similar in nature to what an adult would wear during time of sleep. Any additional layers of clothing are not recommended for infants during time of sleep in the 2022 guidelines. In contrast, the 2016 guidelines recommend dressing an infant in no more than one additional layer of clothing than attire that would be comfortable for adults to wear during sleep.

The 2022 AAP guidelines also reinforce supine sleeping position for infants on a firm, non-inclined surface. Of note, a prone sleeping position for all infants irrespective of medical fragility is also strongly discouraged in the 2022 AAP guidelines, which is not the case in the guidelines from 2016. Also, there is no level of incline or elevated surfaces recommended or supported in the 2022 AAP guidelines, which is another key difference from the 2016 AAP guidelines that promote more leniency in the steepness of surfaces.

In addition, the 2022 AAP guidelines also do not support the use of mattress toppers to make the mattress softer for infants of any age, which is not entirely the case in the 2016 AAP guidelines that also delineate more leniency on when mattress toppers are perceived as safe for use. Notably in the 2022 AAP guidelines, soft mattresses under all circumstances, irrespective of infant sleep position, are strongly discouraged, which also contrasts with the 2016 AAP guidelines that support soft mattress conditions on a case-by-case basis for infants. Furthermore, in the 2022 AAP guidelines, there is increased emphasis placed on assuring that all infant sleep products meet all of the existing federal standards for safe sleep furniture (i.e., cribs, bassinets, play yards, and bedside sleepers).

These guidelines also suggest room-sharing with an infant close to the bedside in a crib or bassinet during the first six months of life. In the 2016 guidelines, there is no timeframe recommended for room-sharing with the infant following birth. Furthermore, the 2022 guidelines thoroughly delineate the perils of bedsharing, which include soft bedding (e.g., pillows, blankets) risks, tobacco exposure, caregiver fatigue, pre-term infant, low birth weight of infant, age of infant less than 4 months of age, among more considerations. Notably, bed-sharing under any circumstances with any caretaker is strongly discouraged in the 2022 guidelines, which is a stark difference from the 2016 guidelines that discourage bed-sharing under specific circumstances (e.g., premature infant with low birth weight, less than 4 months of age). In addition, co-sleeping under any circumstances on any kind of surface is also explicitly discouraged in the 2022 AAP guidelines, which contrast with the 2016 AAP guidelines that present co-sleeping on an adult bed as less hazardous than on an armchair or sofa. Bed-sharing devices are also strongly discouraged in the 2022 guidelines, which is also a key difference from the 2016 AAP guidelines that indicate insufficient evidence for making the case for or against the use of these devices.

Prematurity is certainly a risk factor for sleep-related infant deaths in the Triple Risk Model for SIDS, which is accounted for in the revised AAP guidelines. Decreased gestational age (pre-term infant) has consistently presented as a biological vulnerability that increases SIDS disposition [21]. In addition, decreased gestational age also encompasses low birth weight. The AAP safe sleep guidelines account for both premature and low-birth-weight infants across all recommendations with increased emphasis on the significance of human breast milk consumption for these infants as a facilitator of acquiring nutrients that will protect them against infection [21]. In addition, the 2022 AAP guidelines clearly emphasize that supine sleep positioning is strongly encouraged for both term and preterm infants and integrates content on beginning to transition hospitalized preterm infants from a prone to a supine position at least from 32 weeks of gestational age as a facilitator of safe discharge in the future. With respect to feeding, one of the revised 2022 recommendations promotes increased focus on breastfeeding or use of human milk in other modalities for both full-term and pre-term infants as a SIDS-reduction measure. There are more parameters in the 2022 AAP guidelines on timing of pacifier usage and when to realistically introduce its usage to infants as a SIDS-reduction measure after initiation of breastfeeding whenever possible as well as to encourage its usage during naptime and bedtime. In contrast, there is a suggestion in the 2016 AAP guidelines for consideration on offering a pacifier at naptime and bedtime but not specifically recommending timeframes during the day on when to offer a pacifier to the infant. Of note, recommendations for pacifier practices in the 2022 AAP guidelines are based on increased consistency in findings across research that the use of a pacifier is an evidence-based SIDS reduction measure for infants when given at specified intervals during naptime and bedtime.

In addition, beyond reducing exposure to smoke, alcohol and additional substances during pregnancy and post-partum, the revised 2022 AAP guidelines also suggest reducing exposure to nicotine, marijuana and opioids, especially in light of the growing tobacco epidemic and opioid crisis. Of note, nicotine is found in both traditional and electronic nicotine delivery systems (ENDS). Furthermore, the vaping epidemic emerged more prominently following the 2016 AAP guidelines which do not account for the ever-growing exposure to nicotine across our national and global populations.

In the 2022 AAP guidelines, there are also recommendations for not relying on direct-to-consumer heart rate and pulse oximetry monitoring devices for mitigating or preventing infant sleep-related deaths, given that these devices are solely for consumer wellness and not infant safety. In stark contrast, the 2016 AAP guidelines do not strongly discourage promotion and use of any commercial cardiorespiratory devices for monitoring of infant as a safety measure. Rather, these guidelines indicate that there is no efficacy to be gained from them but do not advocate for avoidance of them.

There are also more parameters suggested for promoting tummy time among infants in the 2022 AAP guidelines from nearly 15 to 30 min daily until the infant turns approximately 7 weeks old. Tummy time is encouraged in the 2016 AAP guidelines without any specifications. In addition, swaddling is strongly discouraged in the 2022 AAP guidelines, especially when an infant begins to roll, likely within the first 3–4 months of life. These guidelines also specify that weighted swaddles or weighted objects contained within swaddlers are not safe and secure for infants in any way. Of note in the 2016 AAP guidelines, swaddling is recommended at individual discretion based on symptomatology and illness-related considerations for infants. Notably, recommendations on swaddling have changed based on findings from research emerging between the publications of both iterations of guidelines. Specifically, the revised guidelines account for findings that have demonstrated consistency in swaddling as a modifiable contributing risk factor for SIDS.

In the 2022 AAP guidelines, there is also increased focus on racial and ethnic disparities that elucidate how black and American Indian/Alaskan Native infants are at nearly two- or three-fold higher risk for SUID than white infants. Furthermore, these guidelines also prioritize the significance of research on interventions to mitigate health disparities in sleep-related infant deaths based on racial, ethnic, and socioeconomic groups. Also, there is also increased focus on safe sleep education, in particular safe sleep practices endorsed and modeled by healthcare providers, media and manufacturers, child care providers, and the Safe-to-Sleep campaign in the 2022 AAP guidelines. Notably, these guidelines support endorsing and modeling SIDS-reduction-related measures from time of prenatal care onward, which contrast with the 2016 AAP guidelines that recommend infant safe sleep education following the time of birth, thereby not accounting for heightening knowledge and awareness on a continuum from prenatal to postnatal phases.

## 3. Deliverers of Information

In one study, some of the messages and videos on recommendations for safe sleep were posted by public health agencies such as the National Institutes of Health and statewide efforts [18]. Sources of content in this study also included the news media on advertising for sleep products such as the bassinet Snoo. Consumers also posted messages with their personal recommendations on safe sleep and SIDS prevention. Additional sources posting tweets included news organizations, universities, and health-related organizations such as the Centers for Disease Control and Prevention, HealthyChildren (the official parenting website of the American Academy of Pediatrics), WebMD, Today’s Parent, and Norton Healthcare. In another study involving examination of whether still images of safe sleep practices on Instagram followed the AAP Safe Sleep guidelines, marketers and decorators posted content pertaining to decorating a new nursery [19]. Sources of content in a different study assessing communication among mothers in a Facebook group were all mothers of infants [20].

## 4. Safe Sleep Recommendations

### 4.1. Positioning of Infants

In two studies, content for risk factors surrounding unsafe infant sleep on Instagram and Facebook included infants sleeping on inclined sleep surfaces, in soft or loose bedding, blankets, pillows, bumpers, stuffed animals, infants sleeping in lateral or prone position, and seated positions, bed-sharing, co-sleeping, and overheated environments [19,20]. One study examined whether content covered recommendations for safe sleep as SIDS-reduction measures and presented information on one or more of the 2016 AAP safe sleep guidelines [18]. In this study, recommendations centered on placing babies to sleep on their backs as well as information on SIDS and safe sleep environments. Of note, this content was posted by HealthyChildren, the parenting website of the AAP, as aforementioned. In another study, the safe sleep recommendations assessed in coverage of still images on Instagram included sleep position (supine), a sleeping infant held by an awake adult, as well as a firm and flat sleeping surface for infants during sleep [19]. Communication among mothers in a Facebook group also encouraged the supine sleeping position for infants [20].

### 4.2. Breastfeeding and Pacifier Usage

There were tweets covered in one study presenting information on how breastfeeding could reduce the risk of SIDS [18]. Content covered in a mothers’ Facebook group presented information that mothers learned from their child’s pediatrician on pacifier usage [20]. There are more parameters in the guidelines on timing of pacifier usage and when to realistically introduce its usage to infants as a SIDS-reduction measure. These times included after initiation of breastfeeding whenever possible as well as during naptime and bedtime.

### 4.3. Tobacco Exposure and Substance Use, Abuse, and Misuse

In the Facebook group, mothers shared information on and avoidance of smoking, which was also in line with the 2016 AAP safe sleep guidelines [20]. In one study, there were tweets on smoking and alcohol use during pregnancy as high-risk health behaviors as contributing factors for SIDS [18]. Cultural factors on Islamic vs. western behaviors pertaining to tobacco and substance use were also delineated across tweets in the same study [18].

### 4.4. Swaddling

There were also tweets and still images on Instagram that yielded content on how swaddling may place babies at risk for SIDS [18,19]. The most prevalent safe sleep furniture covered in still images on Instagram in one study were cribs, bassinets, play yards, or bedside co-sleepers [19]. Additional sleep furniture covered in this study included Moses baskets, adult or child beds, and sitting devices such as car seats, strollers, couches, sofas, the ground, floors, an in-bed co-sleeper, positioner, or infant dock. One study conducted before changes to the guidelines identified tweets centered on safe sleep devices [18]. The specific devices covered across promotional messages on Twitter in this study included initiatives on baby boxes, specifically Baby Box Co, Baby Box University, Finnbin Box, Halo SleepSack, and a baby hammock (not in line with AAP safe sleep guidelines). Another study covering content on Facebook yielded messages from mothers of infants on utilizing baby sleeping devices to mitigate maternal anxiety related to SIDS [20]. These devices were the Rock’n’Play, DockATot, Owlet, Snuza, and Angelcare monitors. Additional devices referenced in the Facebook group included the HALO SleepSack, Merlin’s Magic Sleepsuit, Love to Dream SWADDLE UP, Woombie, and Snuggle Nest. In the same study, there were mixed perspectives from mothers learning information from their child’s pediatrician about whether Rock’n’Plays were in line with safe sleep recommendations [20].

## 5. Infant Loss

One study garnered tweets on narratives of caregivers who lost their infants from SIDS during the month of October, which is Pregnancy and Infant Loss Awareness Month [18]. Narratives included a football player sharing his story on losing an infant and another story about a baby who died in daycare from SIDS in Oregon. In a different study extracting content from a mothers Facebook group, communication among mothers involved ways to support other mothers who had lost an infant from SIDS [20]. This study also yielded content on infant loss attributed to co-sleeping or sleeping in rock’n’plays.

## 6. Social and Emotional Support

One study yielded communication on Facebook between mothers of infants that offered social and emotional support to fellow mothers of infants on a range of topics pertaining to addressing maternal anxiety about SIDS and making decisions for their infants based on what is best for the family irrespective of the safe sleep recommendations [20]. This exchange of communication likely strengthened this group as a support network for mothers of infants.

## 7. Additional Considerations

In one study, mothers felt it would be safer to bed-share and co-sleep with their infants, given the severity of their infant’s reflux [20]. In another study, content in the Facebook group involved information sharing on keeping the house cool without blankets [20]. Two studies uncovered content in tweets on infant health-monitoring devices related to SIDS [18,20]. In one study, tweets disseminated content on potential contributing or causal factors either linked or not linked with SIDS [18]. These factors included the following: vaccines, alteration in serotonin, and brainstem abnormality or cardiac-mediated SIDS. However, in this study, some of the tweets presented factual information clarifying that vaccines are not correlated with SIDS. There was one Australian study that included tweets centered on how brain chemicals are linked to SIDS. Another study uncovered a story published by a newspaper on a doctor who linked hearing dysfunction to SIDS [20]. Tweets also delineated content on how formula did not double the risk of SIDS.

## 8. Assessment of Existing Safe Sleep Content Covered on Social Media

Findings across studies are consistent with several of the recommendations from the AAP 2016 guidelines. Of note, none of the considerations pertaining to perceived contributing or causal factors of SIDS are evidence-based and in turn are not accounted for in the newly revised guidelines. In fact, the majority of these factors covered across the different social media platforms are subjective in nature and allude to misinformation. The revised 2022 guidelines in turn promote more safe sleep education as the basis to mitigate misinformation and disinformation on perceived risk factors for sudden infant death.

Notably, none of the content across the social media platforms in these studies was posted or published by the Safe-to-Sleep campaign. The increased focus on integrating educational content from this prominent longstanding campaign is a significant revision in the 2022 guidelines. It follows that posting content from credible, reliable primary sources could heighten knowledge and awareness as well as mitigate misinformation on a range of considerations pertaining to safe sleep environments for infants.

Of note, none of the content across the social medial platforms supported inclines as a protective factor or acceptable practice in the published studies. This finding is supported by the 2016 AAP guidelines that do not advocate for or against the use of inclines for infants during time of sleep. However, the 2022 AAP guidelines do not support an incline of any kind, thereby endorsing the head of the infant on a bed, and not above the bed.

Coverage of content on thermal conditions in one study involved a recommendation to keep the home environment at a cooler temperature [20]. This finding was in line with the recommendations for thermal considerations delineated in the AAP 2016 guidelines. However, the newly revised AAP 2022 guidelines elucidate more recommendations for thermal conditions among infants which involve increased emphasis on lighter clothing as well as avoidance of hats on infants. It follows that future content on social media could more comprehensively cover all risk factors for thermal instability among infants during sleep, thereby aligning with up-to-date recommendations.

Content across studies covered the perils of tobacco exposure and use as well as substance use, misuse and abuse during both prenatal and postnatal phases, which were in line with the 2016 AAP guidelines. Future coverage on social media could also account for nicotine, marijuana and opioids as evidence-based risk factors for sudden infant death which are delineated in the 2022 AAP guidelines.

Communication between caregivers in one study involved sharing perspectives on how co-sleeping is perceived as safer for infants with reflux [20]. This finding aligns with the nature of the 2016 AAP guidelines that delineate individual discretion in making decisions about co-sleeping under specific circumstances for infants. However, the 2022 AAP guidelines strongly recommend avoidance of co-sleeping. These revised guidelines also suggest room-sharing with the infant close to the bedside in a crib or bassinet during the first six months of life, which was not covered in any of the published studies, although the 2016 AAP guidelines recommend room-sharing without specifying a duration of time. In the same abovementioned study, communication among caregivers also encouraged bed-sharing among infants with reflux [20], which also aligns with the nature of the 2016 AAP guidelines that do not discourage co-sleeping based on individualized circumstances of infants that can include medical conditions. Future coverage of social media content could integrate expansion of risk factors presented in the 2022 AAP guidelines in heightening knowledge and awareness of the constellation of risk factors for SIDS that stem from bed-sharing.

One of the studies supported breastfeeding as a SIDS-reduction measure [18], which is also included in both the 2016 and 2022 AAP guidelines. Two studies presented content on swaddling as a risk factor for SIDS [18,19], which is certainly in line with the 2022 AAP guidelines.

In two of the studies, there was coverage on both support for and against safe sleep devices across Facebook and Twitter [18,20]. The 2016 AAP guidelines support many devices and products approved by consumer safety organizations. However, the 2022 AAP guidelines do not endorse these sleep devices in any way, thereby seeking to mitigate their perceived efficacy and safety.

Given the changes in the AAP Safe Sleep guidelines from 2016 to 2022, it is crucial for social media to account for them in heightening knowledge and awareness on mitigating the perceived safety and acceptability of these devices for infant sleep that have remained longstanding over the years. As aforementioned, the newly revised 2022 guidelines extensively cover recommendations to discourage co-sleeping and utilization of perceived safe sleep devices that are not in line with consumer safety regulations. As part of expanding educational efforts on safe sleep, it is promising that informational, social, and emotional support for caregivers of infants will likely also increase. Oftentimes, trends on social media match with trends across communities on national and global levels. It follows that future coverage on social media of infant safe sleep could extend support for caregivers across the globe.

## 9. Recommendations for Future Research and Practice

Taking everything into consideration, there is an existing paucity of literature on social media coverage of safe sleep considerations for infants to reduce the incidence and prevalence of SIDS worldwide. In fact, this coverage is still in its nascent stages and more timely than ever before in light of its growing visibility and up-to-date evidence-based recommendations in recent times. Further research and practice are warranted, which can encourage including content on social media that is in line with the newly revised 2022 safe sleep guidelines across the most prevalent social media platforms. In addition, based on the prior studies, there is a significant absence of public health agencies posting or publishing content on social media. This suggests that the government and for-profit and non-profit organizations and foundations may not be doing enough to heighten knowledge and awareness of sudden infant deaths attributed to unsafe sleep practices. The vast majority of the knowledge, testimonials and narratives across social media are posted by consumers. None of the content reviewed in the published studies was posted by any credible primary sources promoting these organizational guidelines. It follows that pairing narratives of infant loss with recommendations for infant safe sleep and SIDS prevention could present more compelling and engaging content in heightening a cascade of education on the totality of preventable causes for infant sleep-related deaths.

Future social media coverage on Twitter could also account for content in the Safe-to-Sleep campaign, given that none of the tweets in the sole published study of infant safe sleep on Twitter involved any reference to the campaign. In addition, as previously mentioned, the majority of the content posted on social media was from consumers and was not always consistent with the AAP safe sleep recommendations. It follows that public health agencies in combination with healthcare providers and health systems could potentially partner with consumers to create reliable, credible content that is appealing to caregivers of infants and their networks on mitigating misinformation and disinformation on safe sleep products and devices as well as factors constituting an unsafe vs. safe infant sleep environment.

Of note, there are two existing online support networks pertaining to infant safe sleep: Safe Infant Sleep—Evidence-Based Support Group on Facebook, consisting of more than 162,000 members, and the homepage for the nonprofit organization Safe Infant Sleep on Instagram, which includes nearly 18,000 followers. Healthcare providers could potentially take an active role in participating in communication among caregivers in these virtual spaces that may suggest or allude to misinformation or practices that are not in line with the 2022 AAP safe sleep guidelines. Furthermore, taking this role could help healthcare providers become more visible and reliable experts in the field for all infant caregivers across the globe who are part of these existing online support networks, thereby having a more global reach with disseminating up-to-date knowledge on best practices for infant safe sleep and heightening credibility of content.

Furthermore, in light of the revised 2022 guidelines, which also recommend modeling of safe sleep practices by the media, future work could address the existing coverage of unsafe infant sleep practices and environments on Instagram as well as more social media platforms with increased clarity in discussion and demonstration online as the basis to promote infant safety during sleep to the highest standard. Given the power of social media to disseminate knowledge across the world with increased efficiency, it follows that tapping into social media as a modality of health education and communication on sudden infant death from sleep-related causes could yield promise in reaching and engaging caregivers of infants globally and in turn contribute towards the larger goal of the World Health Organization, United Nations International Children’s Emergency Fund, and Healthy People 2030 in reducing infant mortality on both global and national levels.

## Data Availability

No new data were created or analyzed in this study. Data sharing is not applicable to this article.
